# The Role of Social and Emotional Adjustment in Mediating the Relationship Between Early Experiences and Different Language Outcomes

**DOI:** 10.3389/fpsyt.2021.654213

**Published:** 2021-12-02

**Authors:** James Law, Nathalie Tamayo, Cristina Mckean, Robert Rush

**Affiliations:** ^1^School of Education, Communication and Language Sciences, Newcastle University, Newcastle upon Tyne, United Kingdom; ^2^The Generation R Study Group, Erasmus University Medical Center, Rotterdam, Netherlands; ^3^Department of Child and Adolescent Psychiatry/Psychology, Erasmus University Medical Center, Rotterdam, Netherlands; ^4^Finn Coral Statistical Services, Edinburgh, United Kingdom

**Keywords:** home learning environment (HLE), parental mental health, strengths and difficulties questionnaire (SDQ), pragmatic competence, structural language abilities

## Abstract

**Background:** Studies have highlighted the relationship between early childhood experiences and later language and communication skills on the one hand and social and emotional adjustment on the other. Less is known about this relationship between different types of early experiences and their relationship to different communication skills over time. Equally important is the extent to which the child's behaviour is related to later outcomes affecting the relationship between the child's environment and aspects of their communication development.

**Method:** Drawing on data from 5,000 children in Growing Up in Scotland, a representative sample of children born in 2003. This paper looks are the differential relationships between home learning environment (HLE) (reads books/storeys, engages in painting or drawing, reads nursery rhymes and teaches letter/shapes and parental mental health (PMH) (Depression, Anxiety and Stress Scale (DASS) in the first year of life and both structural language skills (“Listening Comprehension” and “Expressive Vocabulary” subtests of The Wechsler Individual Achievement Tests) and pragmatic competence (The Children's Communication Checklist) at 11 years and explores the extent to which they are mediated by social and emotional adjustment at school entry.

**Results:** PMH was associated with pragmatics but not listening comprehension or vocabulary. By contrast HLE was associated with all three measures of communication. In the final mediated model social and emotional adjustment mediated the relationship between PMH and all three measures of communication. The mediation was statistically significant for the relationship between HLE and both pragmatics and listening comprehension but not for expressive vocabulary. The results are discussed in terms of the relationships concerned and what they tell us about the potential for targeted early interventions.

**Conclusions:** The mediating role of socio-emotional adjustment at school entry points to the need for careful monitoring of children's social and emotional development in primary and middle childhood. Services and policy aimed at improving child outcomes through improving home learning environments must work hand in hand with those responsible for offering support for the mental health, social-emotional adjustment and wellbeing of parents and children from birth and into the school years.

## Background

A range of studies have highlighted the relationship between early childhood experiences and later language and communication skills on the one hand and social and emotional adjustment on the other. These relationships are evident by the time children enter primary school (1, 2) persist during later educational development in school (3), into adolescence (4, 5), and on into adulthood (6–9). Clearly these issues have a direct bearing on adult and teacher perceptions of children when they come into the schools system and potentially have long term consequences for participation in society (10, 11). What is less clear is if and how these experiences and developmental domains are related to one another over the course of early childhood, whether particular aspects of the home environment, for example, are associated with particular subdomains of language and communication development and how social and emotional adjustment might impact on these relationships. For the purposes of this paper language and communication are broad terms used to encompass both the understanding and use of structural aspects of language including the use of vocabulary, the formation of sentences and narratives on the one hand, and the capacity to communicate effectively, often associated with pragmatic competence, on the other. By contrast, social, and emotional adjustment is the perceived reaction of the child to their world commonly described in terms of conduct, activity, emotional responsiveness, and relationships with peers. This starts very early in a child's development but can be difficult to measure in the early years becoming clearer by the time the child starts in school.

### The Relationship of Language to Social and Emotional Adjustment

A key impetus to understanding the nature of the relationship between these variables is that communication skills and social and emotional adjustment are clearly related to one another. This is true within “clinical” samples (12–15), but it is also true in the population as a whole (16–19). The assumption is often that they are related in a linear fashion (20) and that environmental factors play an important role in the development of the relationship between the two (21–23). But cross-domain influences are also conceptualised as developmental cascades, that is the cumulative consequences for development of the many interactions, transactions, and spreading effects across domains which occur in developing systems both concurrently and over time (24, 25). These effects may be direct or indirect, unidirectional or through various pathways, but crucially the consequences are not transient: developmental cascades are closely linked to the course of development.

Although some researchers have suggested that developmental cascades can occur in the relationships between language and communication and social emotional adjustment largely independent of social context (26), others have argued that the relationship between the two should be seen in terms of social adaptation (27). Furthermore, it is widely held that the relationship between the two domains is relatively stable over time (28), although Westrupp, and colleagues have recently suggested that the relationship may be more complex (29). This study shows that the dynamic nature of vocabulary growth trajectories is associated with the development of inattention-hyperactivity and emotional symptoms from childhood to adolescence with literacy as a key mediator. Less attention has been paid to the nature of the relationships between particular aspects or characteristics of the social environment in which the child grows up and different subdomains of language and communication and the potential role that social-emotional adjustment may play in these relationships. Thus it is possible that the child's response to early home learning experiences, may in part be driven by their social- emotional adjustment. A child with poorer social-emotional adjustment may be less able to benefit fully from the learning opportunities available to them and this in turn will affect the relationship between the child's environment and aspects of their communication development.

### Structural Language Development and Pragmatic Competence

Language development is clearly a multifaceted construct. One dimension which has attracted attention in recent years is the difference between the more structural aspects of language (vocabulary, grammar, morphology etc.) (16) and pragmatic competence (understanding of intended meaning, use of language etc.). Studies of the dimensionality of language confirm the emergence of discourse/pragmatics as a construct distinct from vocabulary and grammar by the age of 6–7years (30–32). Pragmatic competence can be defined as “language in use” and its scope can be broadly or narrowly defined. It has proved useful, certainly in intervention research to retain a broad definition encompassing formal linguistic aspects of discourse together with social use of language and inference skills (33). Adams identifies three theoretical models regarding the nature of underpinning knowledge or processing that drive pragmatic language skills: linguistic, social, or cognitive (34, 35). In this study we apply this broad definition of pragmatics, drawing on work in the field of pragmatic language impairments (20, 36–38) and clinical linguistics which considers *pragmatic competence* as emerging from an interaction between the individual's abilities with respect to: (1) interpretation of the literal meaning of the language heard; (2) social cognition, in particular understanding of mental states; (3) the use of contextual cues and general knowledge to interpret language and speaker intentions; (4) the use of prosodic cues to speaker affect and attentional focus; (5) the use of the linguistic context, making connexions between current, and prior elements of the ongoing discourse. Importantly, it is hypothesised that infants' sensitivities to patterns and coordination of vocal timing across adult infant dyads could represent a “proto-pragmatics”: procedural learning which predicts a child's ability to adjust and align the level of contingency of their interactions across communicative partners and from which higher level discourse skills may emerge (39–41). Both structural and pragmatic language abilities have commonly been associated with social and emotional adjustment and recently it has been suggested that pragmatic competence has an important role to play in mediating the relationship between social disadvantage and adolescent behaviour problems (42).

If a distinction between structural language and pragmatic competence is meaningful it would be reasonable to assume that the antecedents of the constituent elements of these two constructs would likely also differ. For example, claims are made that pragmatic language difficulties may be more closely associated with qualities of parent-child relationships—for example in the communication skills of neglected children and their parents' parenting style (43) whereas, by contrast, a child's vocabulary, and comprehension may be associated with the more material aspects of social disadvantage which affect the child's experience (44).

### The Home Learning Environment

Reflecting this, it is relevant to explore two environmental variables which are commonly identified in the literature as potential “risk” factors for language development: namely the home learning environment (HLE) and parental mental health (PMH). Home learning environment captures what it is that parents actually do with their children in terms of stimulation and opportunities provided in the first two or three years of life. Commonly this refers to giving children specific experiences whether through book reading, teaching specific constructs or exposing the child to a breadth of experiences (such as reciting nursery rhymes or painting and drawing) which encourage learning and language development. HLE is recognised to be closely associated with later educational development (45) but has also been shown to be linked to language development in the early years (46–49). Of particular interest is the extent to which HLE is also linked to theory of mind (50) and to socio-emotional adjustment more broadly (48). HLE is not solely determined by social disparities (51) but is clearly related to them (52).

### Parental Mental Health

Clinical levels of poor parental mental health (PMH) has been found to be associated with attachment and child wellbeing and with cognitive development with potential long-term negative sequelae. Thus postnatal depression has been linked to lower communication skills at 12 months (53) and, when the children are older, to internalising (affective), externalising (conduct), and attention problems (54–56) lower academic achievement (57, 58), lower IQ in adolescence (59) and increased school absence. There is also some evidence indicating that treatment for post-partum depression can reduce the negative consequences for the child in the domains of depression and child behaviour although not for cognitive outcomes (60). It is also important to acknowledge that children from mother's with depressive symptoms when the children are younger than 2 years are more likely to have poorer social and emotional adjustment at 3 years of age (61). Additionally maternal depressive symptoms before the child is 5 years has been found to be associated with vocabulary outcomes at 5 years in families living with social disadvantage (62) although the longer term consequences are less well understood Furthermore we are not aware of population based studies that analyse the impact of maternal depressive symptoms on children's pragmatic competence specifically.

### Parental Responsivity

It has been argued that the key common feature across PMH and HLE is the responsiveness of the parent. Children's early language development has been shown to be closely associated with maternal responsiveness and in particular with parenting that is contingent, appropriate and prompt in response to a child's initiations (63, 64). In this way the child hears multiple language models which relate to their focus of attention, thus supporting their mapping of these utterances to meaning through close alignment with the child's communicative intentions. Establishing shared attention and responding appropriately to the child in this way is clearly challenging for a parent experiencing depression or anxiety and it is through this disruption to the responsive parent-child dyad that it is thought that children's language development is hindered in dyads with parents experiencing mental health difficulties. In terms of pragmatic competence, poor parental mental health may disrupt development by affecting the child's ability to develop fully their capacity for inter-subjectivity (i.e., the ability to attribute and understand intentions of others) and subjectivity (i.e., the ability to determine the function of an utterance or communicative act). Very early contingent interactions with the child's primary caregivers even before the child's first words are essential for the development of the child's understanding of both subjectivity and inter-subjectivity (65). Such interactions are likely to be affected in a parent with mental health difficulties and are likely to have a bearing on the child's interaction skills. As the child matures, subjectivity and inter-subjectivity combine to affect the child's understanding of the needs of the listener and their ability to draw social inferences which allow them to make judgement about the appropriacy of the communicative acts whether their own or those of others. PMH and HLE are, of course, likely to be related. Parents with marked mental health difficulties may well find child rearing a challenge and this may be reflected in the home learning environment but there is not necessarily a one to one correspondence between the two and their outcomes may differ. These mechanisms are clearly complex and it is important to consider potential mediators, in part because this helps in understanding the mechanisms involved but also because they help to point to potential intervention targets. One of the most likely candidates, as indicated above is social and emotional adjustment. We know that social emotional adjustment is linked to language but it may also have the potential to protect the child from the effects of early environmental risks.

### The Potential for Intervention

If it is possible to establish that social-emotional adjustment affects the relationship between HLE and/or PMH and language and pragmatics outcomes, it then raises the question of how best to deliver the most appropriate interventions to influence the relevant mechanisms. For example, there is a case for universal interventions to both support parental mental health and promote the child's home learning environment. But such interventions can be difficult to operationalise at a population level because they require good population coverage and the resource to identify the right parents and to support effective interventions. In this case social and emotional adjustment in the early years has the potential to mediate longer term outcomes, although whether any intervention at this stage should be targeted at the child or the parent remains a question as does who should deliver it in the context of the many competing demands placed on schools and teachers (66). But this then raises the question of the intervention outcomes. In the model proposed here it is the child's social and emotional adjustment that is key to their cognitive outcome, in this case measured by the child's structural and pragmatic language skills. One alternative, of course, is simply to focus on the latter skills targeting low achievers in those skills early in their development. The reality is that services will likely need to function at multiple levels, at a public health level focusing on public awareness of the importance of parent/child interactions and of the home learning environments, at a targeted selected level (covering “at risk” populations in particular families living with poverty and/or with a history of mental health difficulties) and at a targeted indicated level for those where the social- emotional adjustment and/or language development is not developing as expected. Another alternative is to focus on the within-school experience of children and paying greater attention on the relationship between teacher and pupil. Recent evidence suggests that promoting better within-class relationships in the primary school period are associated with more positive communication skills in adolescence (25, 67).

### Hypothesis

*The current analysis* is broadly framed with the family stress model (68). While stresses affect children across all domains we would argue that it is often their communication skills where the effects are paramount. Clearly economic stress underpins many of the deleterious effects on parents and children (69) but stress is also a function of the individual's response to those pressures. Clearly parental mental health is a manifestation of that response for many.

**Figure 1:**
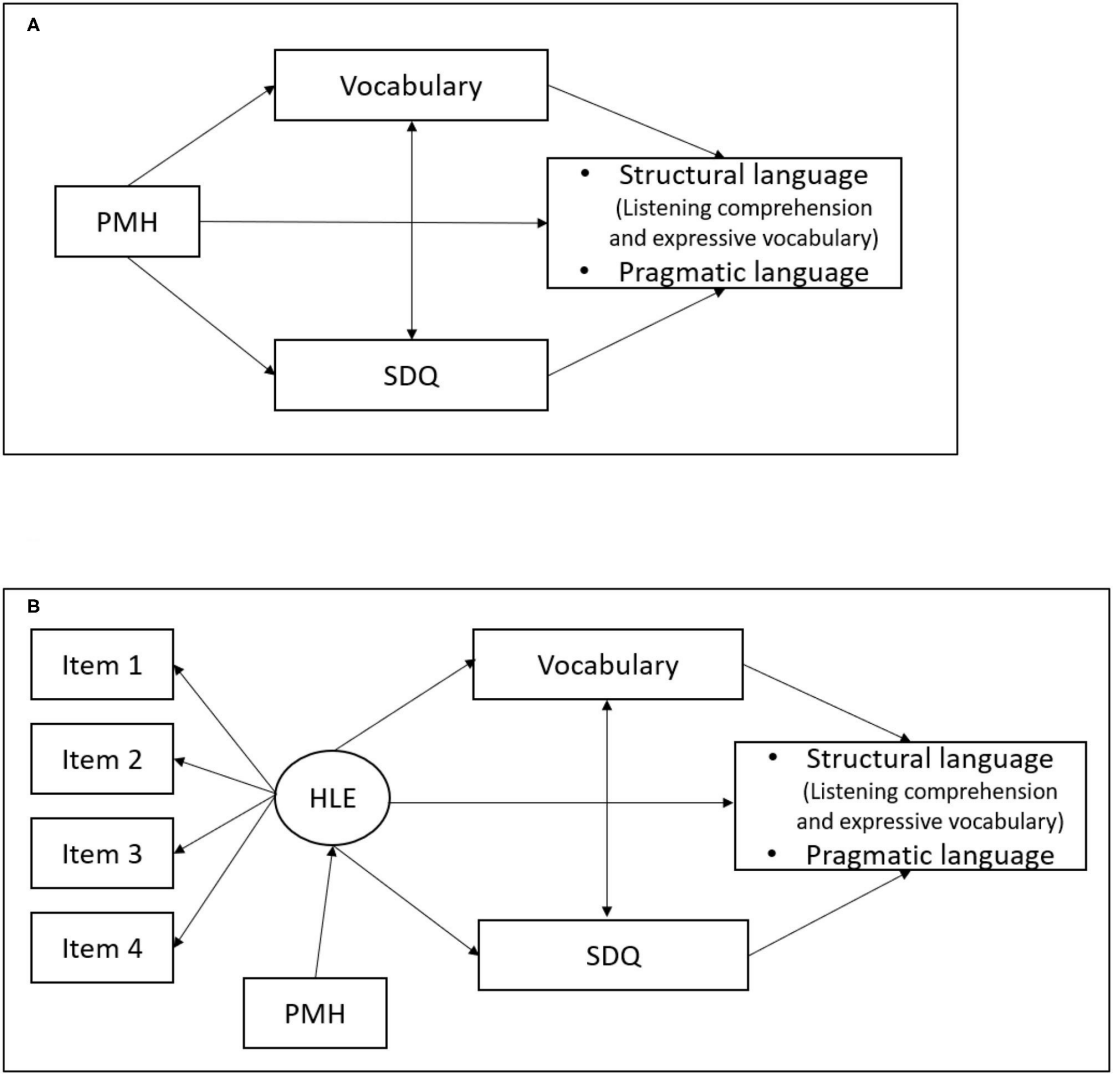
Outline of the mediation models. **(A)** Model with parental mental health as exposure. **(B)** Model with home learning environment as exposure.

The study explores the relationship of two separate characteristics of children's early environment (HLE and PMH) to the separate language subdomains of structural language and pragmatic competence (70–72) examining the role that social and emotional adjustment plays in this relationship (73, 74). We consider structural language and pragmatic competence outcomes at age 10 years: a particularly important age marking the transition from primary to secondary school and, with it, additional demands on children's social, emotional, and academic functioning. Those who are vulnerable in these domains at this point may find these demands outstripping their abilities, causing issues with self-esteem, educational engagement and peer relationships (75–78). In the present analysis we hypothesise firstly that HLE and PMH will be associated with different degrees to structural language and pragmatic competence at this point and secondly that a child's socio and emotional adjustment at school entry will demonstrate the potential to mediate these relationships. Mediators may, of course, enhance a relationship, buffer it or be antagonistic (reversing the effect of the predictor). These relationships are conceptualised in Figures 1A,B below.

To address complex questions of this nature it is important to employ data from large-scale nationally representative longitudinal cohorts rather than clinical studies to avoid potential over-estimates of the relationships of interest. To date it has been very difficult to do this because, although variables associated with HLE and PMH and indeed social and emotional adjustment, are commonly found in such cohorts, measures of structural and pragmatic language competence are not. The focus of this study is the Growing Up in Scotland study (GUS) which includes all five variables of interest. Previous research from GUS (79–82) has demonstrated the often stark inequalities at age three in both children's cognitive ability and their social and emotional development from early through to middle childhood.

### Research Questions

Research question 1: *To what extent are the home learning environment and parental mental health associated with structural and pragmatic language skills at the end of primary school?* We hypothesise that HLE items and PMH scores influence later structural and pragmatic language outcomes both directly and also indirectly through social-emotional adjustment.
Research question 2: *To what extent do emotional and behavioural difficulties mediate the relationship between parental mental health and home learning environment on the one hand and structural and pragmatic language difficulties on the other?*

## Methods

### Participants

The data in this study are taken from the Growing Up in Scotland (GUS—https://growingupinscotland.org.uk/) Study a birth cohort commissioned in 2003 by the then Scottish Executive Education Department, and managed by ScotCen Social Research (ScotCen). GUS is a large-scale longitudinal research project aimed at tracking the lives of several cohorts of by a wide-ranging purpose, the principal aim of the study is to provide information to support policy-making in Scotland, but it is also intended to be a broader resource that can be drawn on by academics, voluntary sector organisations, practitioners and other interested parties.

The study population was derived from child benefit records (at that time this was a universal benefit with around 97% of eligible families in receipt of this benefit in Scotland). Stratified cluster sampling was used to derive a nationally representative sample. Primary sampling units (PSUs) were first created by aggregating data zones (small, relatively socially homogeneous, geographical areas of adjacent postcodes with 500–1,000 residents) in order to give an average of 57 births per sampling unit per year based on the previous 3 year's birth rates in the relevant data zones. PSUs were then stratified according to local authority area and then Scottish Index of Multiple Deprivation score. One hundred and thirty PSUs were then randomly selected across strata to ensure a representative mix of areas in terms of socioeconomic status and local authorities. To date there have been eight sweeps of the GUS cohorts as follows Sweep 1 (0–1 years); Sweep 2 (1–2 years); Sweep 3 (2–3 years); Sweep 4 (3–4 years); Sweep 5 (4–5 years); Sweep 6 (5–6 years); Sweep 7: (7–8 years), and Sweep 8: (9– 0 years**)**. In these analyses the predictors are identified at sweep 2, the mediator at sweep 5 and the outcomes at sweep 8. It is important to emphasise that all families with children receive child benefit in the UK and the resulting sample is thus representative of the country as a whole. The total number of main carer interviews completed in sweep 8 was 3,148 and the number of child interviews was 3,087 and of these 2,608 were included in the structural equation models reported below.

Clearly, by definition, there will be a subgroup of children who are likely to have specific difficulties with pragmatic competence arising from a neurodevelopmental condition such as an autism spectrum disorder (ASD) or social communication disorder (SCD) (36). GUS does not include information about diagnoses, rather parents are asked whether the child has any formal diagnoses. Fortunately, this question is asked at 8 years of age and one can be reasonably sure that children with this type and level of need would have been identified at the point and the parents are likely to know this. Neither would necessarily be true of the parents of younger children. Accordingly 29 children out of a total of 2,791 children at age 8 were identified. This is equivalent to a prevalence of 1%—in line with UK published prevalence estimates (83) although rather lower that the most recent parent reported estimates from the US (84). This group was then taken out of the data set used for further analysis.

### Predictors

#### Home Learning Environment

Items were selected which reflect aspects of the HLE (which also represented a spread of responses). Four variables were identified in the data which captured in a similar fashion (in terms of question formation) four separate activities which would be considered appropriate ‘elements' of the home learning environment. These are “How many days **in the last week** have you:-

- read books/storeys
- engaged in painting or drawing
- read nursery rhymes
- taught letter/shapes.

Responses classed as “not applicable” indicates that the associated frequency question did not apply, as they did not undertake that activity, and hence it was not relevant to them (following personal exchange with Paul Bradshaw, Director of GUS). Initially we tried to create a composite measure in two ways; averaging the responses over those items, and a factor analytical approach. The internal consistency was poor of the averaging and the factor analysis had low Kaiser-Meyer-Olkin adequacy (0.6). Thus it was decided to use the items individually in the mediation analysis. The HLE variables were entered as continuous with scores on a seven point likert scale.

#### Parental Mental Health

Six items from the Depression, Anxiety and Stress (DASS) scale (85) were included in the self-completion section of the interview. DASS in full form is available in a 42-item, or 21-item scale. The 6 items included in GUS comprised 3 measuring stress, and 3 measuring depression. The included items were as follows:-

A1—I found myself getting upset by quite trivial things (stress) A2—I found it difficult to relax (stress)
A3—I felt that I had nothing to look forward to (depression) B1—I felt sad and depressed (depression)
B2—I found that I was very irritable (stress)
B3—I was unable to become enthusiastic about anything (depression).

The stress and depression items can be combined to create separate stress and depression scales and standardised versions of these scales (z-scores) averaged to produce a single scale measuring evidence of negative emotional symptoms in the respondent (86). It was this negative emotional symptoms scale which was used in the analyses.

### Covariates

Six covariates were identified:-

- Age in months at wave 8 (10 year old) assessment
- Gender
- Birthweight
- Non-verbal IQ at 3 years (BAS Picture Similarities).
- Education of main respondent recorded in GUS sweep 1.
- Expressive vocabulary at 5 years (BAS Naming Vocabulary).

### Outcomes and Mediator

#### Pragmatic Competence

Sweep 8 includes a parent report measure of children's communication which uses selected items from the “Pragmatics” subscale of the Children's Communications Checklist (CCC2) (87, 88). Items from the CCC 2 have also previously been used in the Avon Longitudinal Study of Parents and Children (ALSPAC http://www.bristol.ac.uk/alspac/). The CCC 2 consists of nine subscales to measure children's communicative ability: Speech, Syntax, Initiation, Coherence, Conversation, Context, Rapport, Social Behaviour, and Restricted interests. The pragmatics composite of the CCC is based on Scales C to G of the CCC, and reflect the components of pragmatic competence described above (38): namely, initiation (e.g., “Talks repetitively about things that no-one is interested in”); Coherence (e.g., “Would have difficulty in explaining to a younger child how to play a simple game such as ‘snap”'); conversation (e.g., Make frequent use of expressions such as “by the way,” “actually,”“you know what?,” “as a matter of fact,” “well you know,” and “of course”); Use of context (e.g., tends to repeat back what others have just said); and Rapport (e.g., Doesn't seem to read facial expressions or tone of voice adequately and may not realise when other people are upset or angry) (89). Twenty five items were asked of the GUS cohort child's main carer as part of the self-completion section. The CCC has a reported interrater reliability of 0.8 across the scales (range, 0.62–0.83) with Cronbach's alpha of 0.867 for one rater and 0.797 for a second. The clinical validity of this scale has been shown to be good, using a threshold of 132 or below to indicate pragmatic language impairment. A normative study gave a mean of 153.7 and SD of 6.5.10.

#### Structural Language

The Wechsler Individual Achievement Tests, 2nd Edition (WIAT-II) http://www.pearsonclinical.co.uk/ Psychology/ChildCognitionNeuropsychologyandLanguage/ ChildAchievementMeasures/WechslerIndividualAchievement Test-SecondUKEdition (WIAT-IIUK) was carried out at sweep 8. Wechsler (90) The WIAT-II measures cognitive skills on a series of continuous scales. The assessments carried out with the GUS children were adapted for use in a survey setting, and modified to be administered *via* Computer Assisted Personal Interviewing (CAPI). Critical to the present analysis Oral language subtests “Listening Comprehension” and “Expressive Vocabulary.” For listening comprehension the child is asked to select a picture that matches a sentence and for expressive vocabulary the child is asked to generate a word that matches a picture and oral description. We use the term “structural language” to denote both expressive and receptive elements of language which tap into semantic elements of language, different from the much more contextually determined pragmatic language described above. Clearly, to some extent, vocabulary underpins more sophisticated structural language elements and, while they often appear closely associated in young children, differences between them may emerge over time (91). These elements could be differentiated further by adding more detailed consideration of morphology, syntax and discourse etc. in future analyses but this was not possible in the available dataset.

#### The Mediator—Social and Emotional Adjustment

The mediator variable was the Strengths and Difficulties Questionnaire (SDQ), a 25-item checklist of a child's social and emotional adjustment (92, 93). This can be used from 2 years but becomes increasingly stable over time.

The main carer SDQ provides a Total Difficulties Score, which is the sum of scores for the emotional, conduct, hyperactivity, and peer problems subscales. Each of the 5 scales of the SDQ are scored from 0 to 10, and one can add up 4 of these (emotional, conduct, hyperactivity, and peer problems) to create a total difficulty score (range, 0–40). There is also a score for the children's strengths—the Prosocial score—which, like the others, has a maximum score of 10 but works in reverse, with a high score indicating more pro-social behaviours, but this is not included in the total difficulties score and was not therefore included in the present analyses. For each question, the respondent is required to say whether a statement is “not true,” “somewhat true,” or “certainly true.” The internal consistency of the SDQ is relatively high (mean Cronbach's alpha 5.73) as is the retest stability after 4–6 months (mean: 0.62).

### Analytic Approach

Descriptive statistics (means and SD) and inter-correlations between measures precede the meditational analysis. Figures 1A,B provide the relationship to be explored between HLE and PMH, at sweep 2 as the explanatory variable and the language outcomes (pragmatics, expressive language and listening comprehension—structural language) at sweep 8, in the presence of the potential mediating variable, social and emotional adjustment measured, at sweep 5. These relationships were assessed with six Structural Equation Models (SEM) to account for the measurement of the HLE as a latent construct, (94) the complete reference is before the references of the manuscript. A path with an earlier measure of expressive language was specified in the model, although we did not estimate a mediated effect through this path. The analysis was performed with Lavaan (95), using maximum likelihood estimation with robust standard errors to account for missing data.

Models were adjusted for maternal education, child sex, birth weight, non-verbal IQ and age at outcome assessment. Models with home learning environment as exposure are further adjusted for parental mental health. Mediation was tested using standard techniques commonly associated with Baron and Kenny (96), further developed more recently by Hayes (97, 98). Throughout the analyses, sampling weights were employed to adjust for the oversampling in the GUS.

### Ethical Approval

The initial sweep of data collection was subject to medical ethical review by the Scotland “A” MREC committee (application reference: 04/M RE 1 0/59). Up until and including sweep 8, subsequent annual sweeps have been reviewed *via* substantial amendment submitted to the same committee. Sweep 9 and 10 were subject to ethical review by the NatCen Research Ethics Committee.

## Results

The characteristics of the population are provided in Table 1. Half of the child population were males. Parental education was reported to be a vocational qualification in 37.5% of the respondents, 27, 6% a degree and 17.1% a standard grade.

**Table 1:** Characteristics of the study population.

	**Mean(SE)**	**Sweep**
Birth weight in grammes	3384.64 (14.76)	1
Composite DASS score	0.03 (0.02)	2
Picture Similarities T-Score	50.09 (0.30)	3
BAS Naming vocabulary T-Score	54.01 (0.24)	5
SDQ: Total difficulties score	7.63 (0.14)	5
Study child's age at interview (months)	122.23 (0.11)	8
Pragmatics total	87.61 (0.25)	8
Listening Comprehension Raw Score	27.05 (0.14)	8
Expressive Vocabulary Raw Score	7.96 (0.08)	8
Books/storeys in last week	6.25 (0.05)	2
Painting or drawing in last week	3.89 (0.05)	2
Nursery rhymes in last week	5.04 (0.06)	2
Letters/shapes in last week	3.28 (0.07)	2
**Sex of the child**	**%** (**N)**	1
Male	50.87 (1,317.72)	
Female	49.13 (1,272.68)	
Total	100 (2,590.39)	
**Parental highest education**		1
Degree or equivalent	27.59 (713.92)	
Vocational qualification below degree	37.47 (969.51)	
Higher grade or equivalent	8.28 (214.11)	
Standard grade or equivalent	17.08 (441.86)	
Other	0.44 (11.28)	
No Qualifications	9.14 (236.55)	
Total	100 (2587.23)	

Evaluating the correlation between PMH and HLE we found that PMH is associated with frequency of sharing books and storeys (−0.08), painting or drawing (−0.03) nursery rhymes (−0.07) and letters and shapes (−0.02). The CCC2 score is associated with listening comprehension (0.23) and with expressive vocabulary (0.19). By contrast, the association between listening comprehension and expressive vocabulary was much higher (0.82).

The univariable association of each language outcome (pragmatics, listening comprehension and expressive vocabulary) and modelling the independent variables, covariates and mediators as predictors are presented in Table 2. Parental mental health was negatively associated with pragmatics (Beta = −2.4, 95% CI [−3.1, −1.8], *p* < 0.001), to listening comprehension (Beta = −0.3, 95% CI [−0.5, −0.02], *p* < 0.001), but not to expressive vocabulary (Beta = −0.1, 95% CI [−0.2, −0.1], *p* 0.3). Conversely, HLE was associated with pragmatics (Beta = 0.7, 95% CI [0.4, 0.9], *p* < 0.001), with listening comprehension (Beta = 0.5, 95% CI [0.3, 0.7], *p* < 0.001) and with expressive vocabulary (Beta = 0.2, 95% CI [0.1, 0.3], *p* < 0.001).

**Table 2:** Univariable effects of the predictors on the outcomes at 10 years (including the effects of the mediator) co-varying for age at 10 year assessment, gender, non-verbal IQ (Picture similarities), birthweight and education of main respondent.

	**Pragmatics**		**Listening comprehension**		**Expressive vocabulary**	
	**B(95%CI)**	**Sig**.	**B(95%CI)**	**Sig**.	**B(95%CI)**	**Sig**.
Gender (Male)	−2.19 (−3.14, −1.25)	<0.001	0.24 (−0.17, 0.65)	0.25	−0.02 (−0.24, 0.19)	0.84
Birthweight (Kg)	0.89 (0.16, 1.62)	<0.05	0.69 (0.32, 1.02)	<0.001	0.24 (0.03, 0.45)	0.027
Picture Similarities T–Score	0.16 (0.11, 0.20)	<0.001	0.12 (0.09, 0.14)	<0.001	0.05 (0.04, 0.06)	<0.001
Childs age at interview (months)	−0.11 (−0.24,0.01)	0.08	0.11 (0.06, 0.17)	<0.001	0.06 (0.03, 0.09)	0.001
BAS Naming vocabulary at 5 T-Score	0.18 (0.14, 0.21)	<0.001	0.12 (0.30, 0.39)	<0.001	0.06 (0.05, 0.07)	<0.001
Parental Highest Education (Ref: No Qual.)
Degree	8.49 (6.59, 10.38)	<0.001	3.98 (3.06, 4.89)	<0.001	1.68 (1.17, 2.19)	<0.001
Vocational qualification below Degree	4.60 (2.67, 6.54)	<0.001	2.43 (1.54, 3.33)	<0.001	1.03 (0.53, 1.53)	<0.001
Higher Grade	6.02 (3.81, 8.23)	<0.001	3.04 (1.94, 4.15)	<0.001	1.29 (0.69, 1.89)	<0.001
Standard Grade	1.51 (−0.91, 3.93)	0.21	1.87 (1.01, 2.73)	<0.001	0.74 (0.19, 1.29)	<0.01
Other	4.50 (0.79, 8.22)	<0.05	3.58 (0.01, 7.16)	0.05	1.23 (−1.07, 3.54)	0.28
Composite DASS score (PMH)	−2.41 (−3.06, −1.77)	<0.001	−0.28 (−0.54, −0.02)	0.04	−0.07 (−0.22, 0.09)	0.39
Home Learning Environment						
Books/storeys in last week	0.69 (0.40, 0.99)	<0.001	0.48 (0.32, 0.65)	<0.001	0.20 (0.12, 0.29)	<0.001
Painting or drawing in last week	0.43 (0.21, 0.64)	<0.001	0.13 (0.04, 0.21)	0.005	0.09 (0.05, 0.13)	<0.001
Nursery rhymes in last week	0.74 (0.55, 0.92)	<0.001	0.23 (0.14, 0.32)	<0.001	0.12 (0.07, 0.17)	<0.001
Letters/shapes in last week	0.19 (0.03, 0.35)	0.02	0.18 (0.12, 0.24)	<0.001	0.08 (0.05, 0.12)	<0.001
Total difficulties score (SDQ)	−0.94 (−1.04, −0.84)	<0.001	−0.19 (−0.24, −0.14)	<0.001	−0.07 (−0.10, −0.05)	<0.001

**Figure 2:**
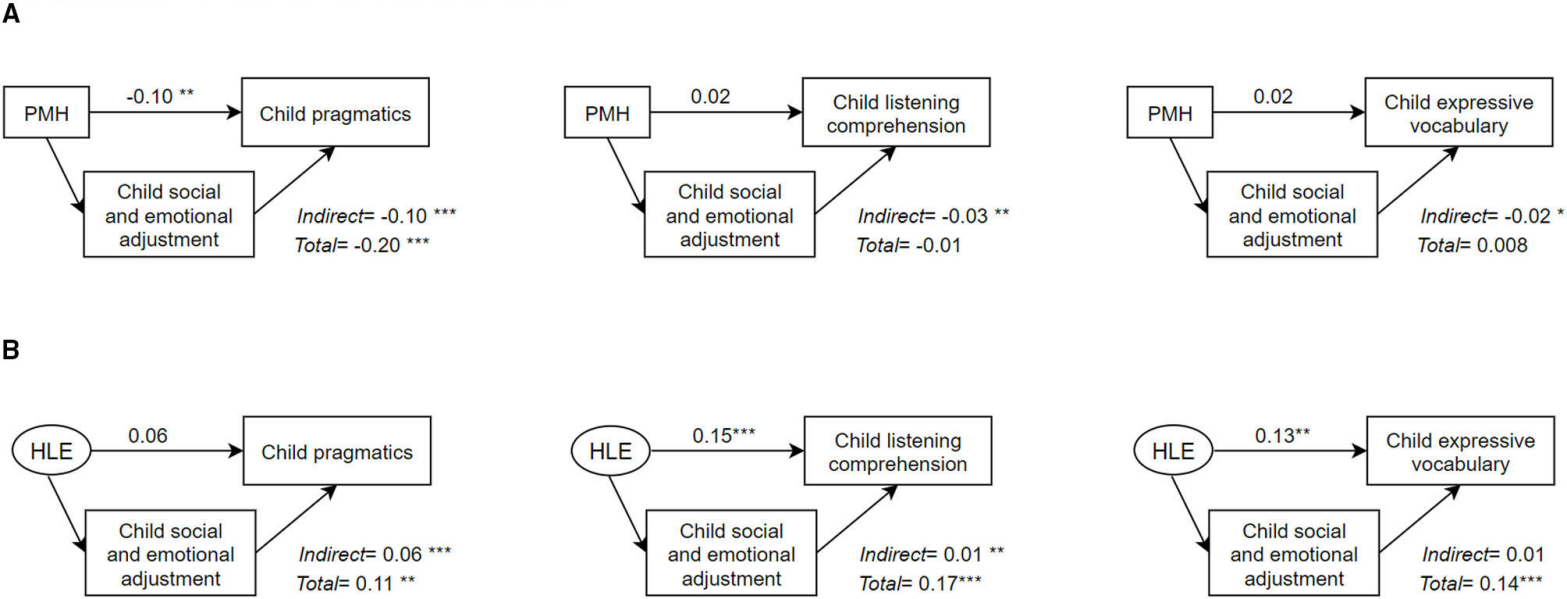
Mediated effect of child social and emotional adjustment in the association between parental mental health or home learning environment and child language ability. The estimates correspond in Figure 1 to the direct paths and the indirect paths through child social and emotional adjustment. Each mediation model corresponds to one model. Models are adjusted for maternal education, child sex, birth weight, non-verbal IQ and age at outcome assessment. Models with home learning environment as exposure are further adjusted for parental mental health. Standardised coefficients are presented. *p < 0.05, **p < 0.01, ***p < 0.001. **(A)** Mediation models with parental mental heath as exposure. **(B)** Mediation models with home learning environment as exposure.

The estimates for the mediated models are presented in Figure 2 and the complete output in Supplementary Table 1. These estimates correspond to the direct paths and the indirect paths through child social and emotional adjustment in Figure 1. PMH was associated with child pragmatics (total effect, Beta = −0.2, 95% CI [−0.3, −0.1], *p* < 0.001), but not associated with listening comprehension (total effect, Beta = −0.1, 95% CI [−0.1, 0.03], *p* < 0.6) and expressive vocabulary (total effect, Beta = 0.008, 95% CI [−0.1, 0.1], *p* < 0.001). Nonetheless, there was a significant mediated effect through child social and emotional adjustment in the association of parental mental health and child pragmatics (indirect effect, Beta = −0.1, 95% CI [−0.1,-−0.1], *p* < 0.001), listening comprehension (indirect effect, Beta = −0.03, 95% CI [−0.1, −0.01], *p* < 0.01 and expressive vocabulary (indirect effect, Beta = −0. 02, 95% CI [−0.03, −0.002], *p* < 0.05).

For the HLE models the factor loadings of the HLE items were constrained to be equal. The models fit were: Comparative Fit Index (CFI) >0.95 and Root Mean Square Error of Approximation (RMSEA) < 0.6. The HLE was associated with child pragmatics (total effect, Beta = 0.1, 95% CI [0.03, 0.2], *p* < 0.001), as to listening comprehension (total effect, Beta = 0.2, 95% CI [0.1, 0.3], *p* < 0.001) and expressive vocabulary (total effect, Beta = 0.1, 95% CI [0.1, 0.2], *p* < 0.001). Additionally, there was a significant mediated effect through child social and emotional adjustment in the association of HLE and child pragmatics (indirect effect, Beta = 0.1, 95% CI [0.03, 0.1], *p* < 0.001) and listening comprehension (indirect effect, Beta = 0.01, 95% CI [0.004, 0.02], *p* < 0.01 but not a significant mediated effect on expressive vocabulary (indirect effect, Beta = 0.01, 95% CI [−0.001, 0.02], *p* = 0.08).

## Discussion

In this study using data from a population-based prospective cohort, we examined the association between parental reported depressive symptoms and stress; the home learning environment at child age 2 years and child language development at age 8 considering the pragmatic and structural aspects of language. We found a negative association between parental mental health and pragmatic language in the child. For the HLE we found a positive association with pragmatics and the listening comprehension component on structural language in the child, suggesting that, although there is a small negative association in each case, i.e., the higher the HLE scores the lower the parental mental health problems score. Nonetheless these relationships are relatively modest even though it might be argued that the concepts potentially overlap considerably. So while HLE may be affected by parental mental health it is by no means necessarily the case. The same issue arises with the outcome variables (structural and pragmatic language) being correlated. It is probably less of an issue with the association between receptive and expressive language which are known to be closely related. It might be argued that they were really capturing the same construct.

*Research question 1: To what extent are the home learning environment and parental mental health associated with structural and pragmatic language skills at the end of primary school*.

In answer to the first question it is clear that there do appear to be different levels of association between our predictors and both structural and pragmatic language outcomes at 10 years. Parental mental health is directly associated with pragmatics which is a novel finding. Contrary to previous research (53), we did not find an association (total effect) between parental mental health and structural language. We hypothesise the differences might be because our sample is from the general population and not clinical, therefore the prevalence of parental and child problems are lower and more difficult to find. Whereas the HLE is associated with both pragmatics and the listening comprehension component of structural language. Social and emotional adaptation at 5 years is consistently related to all three outcomes, reflecting the recognised pattern of the association between social-emotional adjustment and language, this association is significant independently of child expressive vocabulary at age 5. This suggests that structural and pragmatic language measured using these specific assessments are not one and the same thing, especially as the child reaches the end of primary schooling (30–32).

*To what extent do emotional and behavioural difficulties mediate the relationship between parental mental health and home learning environment on the one hand and structural and pragmatic language difficulties on the other?*

The results suggest that social and emotional adjustment mediates the relationship between parental mental health and pragmatics as to the structural component of language. The mediation effect for the home learning environment is comparatively small and for pragmatics and only to the listening comprehension component of structural language. It is important to note that these relationships hold once adjusted for the covariates, age, gender, previous expressive vocabulary, non-verbal IQ (Picture similarities), birthweight and education of main respondent and mental health in the HLE models.

### Implications for Theory

We identify three implications. The first is that PMH and HLE independently impact upon pragmatics suggesting that the interaction between parent and child affect the capacity to interact through language. Part of this is mediated through the child's social and emotional adjustment. Secondly there seems to be no direct association between PMH and structural language but PMH does relate to social and emotional adjustment and through it structural language. This might be an explanation for what we see in clinical samples where the link between social and emotional adjustment and language are well recognised. Thirdly HLE is associated with listening comprehension which is consistent with previous literature. The findings underline the complexity of describing the nature and quality of parent child interactions and their effects on child development. The *specific* impacts of PMH on pragmatic competence and of book reading on structural language suggest that there is much still to learn about which *specific* aspects of contingent and responsive interactions underpin inter-subjectivity (i.e., the ability to attribute and understand intentions of others) and subjectivity (i.e., the ability to determine the function of an utterance or communicative act) and ‘proto-pragmatics' (40) and which are crucial for vocabulary and grammar acquisition. Indeed, there is a good case for arguing that pragmatic skills anticipate the development of vocabulary (39) and may be especially sensitive to parental mental health difficulties experienced in the first year of life (as indicated above). Whether these skills are actually manifestations of specific inherited characteristics remains out of the reach of an essentially “social” data set, albeit large ones such as GUS. By contrast educational experiences such as those captured by HLE perform a rather different function in shaping a child's experience of language. The former may lead to the development of a spiral or cascade of poorly functioning social interaction that are evident in social and emotional adjustment and in the pragmatic language skills in this study, whereas the latter affects the symbolic skills underpinning language, limiting comprehension and the development of vocabulary and by inference more advanced structural language skills.

### Implications for Further Research

Further research would be warranted in a number of related research directions. The first is to explore the different subscales of the SDQ in the meditational role to establish whether any of the subscales were especially salient. Earlier analysis suggested that only the prosocial scale seemed to operate in a different way to other subscales but other studies have specifically sought to tease out the difference for the hyperactivity and emotional symptoms subscales (29). Another dimension that is often cited in relation to both social and emotional adjustment and language is gender. In the present analyses we included gender as a covariate in establishing the relationships identified here. Because boys and girls characteristically display different developmental profiles for social-emotional adjustment and girls are thought to consistently out-perform boys in terms of language outcomes, [although see (99) for an alternative cohort based view] one might anticipate that gender might operate as a moderator of behaviour as an outcome.

Similarly, it would be interesting to follow Westrupp et al. and look at *trajectories* of both aspects of communication. But there would be limited potential for the former because, while there are language measures at different time points the measures are not the same. The latter might be possible but only if one would be prepared to accept the simplifying assumption, made by some, that pragmatic skills are captured by the pro-social scale of the SDQ. Our view on this would be that the level of detail in the CCC2 far outweighs that in the SDQ prosocial scale which would not warrant such an assumption.

The association which we have seen here between PMH reported early in the child's life and social and emotional adjustment with later child outcomes is obviously important as far as the child is concerned but it would be wrong to assume that the potential consequences are significant only for the child. The heritability of mental health problems rather suggests that the pragmatic competence of the parents ought to be a focus of research interest to allow professionals to create interventions which are of value to parent and child. There is probably only limited value to target individual children when they spend so much of their time in a communicative context which may not be helping develop their pragmatic language skills.

HLE has long been a focus of intervention efforts as witnessed by the current campaigns in the UK (100, 101) but care has to be taken to avoid assuming that it is the activity alone which is important rather than the communication and learning experiences associated with those activities. An interesting finding which requires further inspection is as why reciting nursery rhymes appears to be the only aspect of HLE which remains related to both pragmatic competence and structural language.

A further important area for research is to consider the role of social disadvantage in these relationships, not simply adjusting it out of the models but rather considering whether it has the potential to moderate the mediational effect of socio-emotional adjustment. Perhaps the parents in socially disadvantaged families may have less available “resource” with which to compensate for or modify their interactions in response to poorer socio-emotional adjustment in their child a suggestion which would be supported by the family stress model. But it may be language trajectories have an independent dynamic with socio-emotional development which is not solely driven by stress and are linked to attainment such as literacy skills (29).

The complex nature of contingent interactions between parent and infants and their unfolding influence over childhood clearly remains a fertile area for future research. Advances in analytical methods and the availability of rich data sets of videoed parent/child interaction for example in the Avon Longitudinal Study of Parents and Children (ALSPAC) (http://www.bristol.ac.uk/alspac/) in the UK and the National Education Panel (NEPS) in Germany (https://www.neps-data.de/en-us/home.aspx), hold promise for the delivery of new insights in the near future.

### Implications for Practise

There is a good case from the evidence in this paper to ensure that both structural language and pragmatic competence are monitored in middle childhood. These skills clearly are sensitive indicators of aspects of the child's development which are closely linked to earlier developmental and risk profiles. The fact that earlier risk factors (conventionally the province of early health workers) link to social and emotional adjustment at school entry at communication outcomes at the end of primary school warrants attention. This chimes with the findings of McKean et al. (102) which suggest that children with below average language skills *and* poor social-emotional adjustment at school entry are particularly vulnerable to very poor outcomes by 11 years of age (102). In addition to identifying individuals a risk the mediational relations examined here, implying some degree of causation and thus have the potential to inform the development of interventions (103). Thus there is clearly an inference to be drawn here about providing interventions targeting both the mental health of the parent and interventions supporting the Home Learning Environment in the first year of the child's life and the social and emotional skills of the child, perhaps supported by parental mental health interventions. The data in this study suggest that the timing of such child interventions should coincide with school entry but clearly children may be manifesting social and emotional adjustment difficulties before this and may continue to do so thereafter. Clearly these should involve both parents and teachers. There is some suggestion that language interventions may have a bearing on behavioural outcomes (104) and there have been developments in interventions to promote pragmatic skills (35), but there is a long way to go to give a clear answer to the question as to whether behavioural interventions can alter the mechanisms discussed above. It is another matter again as to whether they should be universal or targeted and whether they should focus on parent or child or indeed both. For example, it may well be that the pragmatic skills of the parents, especially those with any history of mental health difficulties warrant attention. These data suggest that there is an urgent need to address the behaviour of children as they enter the school system but the outcomes need to go beyond the social and emotional adjustment of the child to the communication outcomes discussed in this paper which are so essential for effective functioning in adolescence. But whether these interventions should be targeting those behaviours in individual children or promoting better pupil/teacher classroom interactions as a way of improving later communication skills warrants further empirical investigations (25, 67).

### Study Limitations

As with most population studies such as GUS, assessment detail often has to be offset against sample size. The larger the sample and the broader the range of domains covered the less time is available for detailed individual assessments. In the case of the research questions posed in the current study however, we would argue that the GUS cohort manages this trade off relatively well. Nevertheless there are, of course, other variables which may play a role, and where detailed assessments might enhance the analysis (e.g., for example executive function). There is also the potential concern of relying too heavily on parental report for data although such questionnaires have been found in other studies to be relatively robust (105). The issue of correlations due to shared reporter bias is a sensible concern. The parent is the respondent for all the information here except the language assessments and there is a risk that results maybe inflated for this reason. If this was the case one would expect to see higher associations between parental scores than the test scores. Although this is true when we look at the report of parental mental health and the pragmatic language abilities the profile varies considerable when we look at the HLE at a univariable level and the relationships vary considerably in the final mediated analysis.

Nevertheless it is an important consideration to bear in mind especially in the case of pragmatic language skills which are, by definition, likely to be reported by another commenting on a child's communicative competence. Clearly these relationships warrant further explanation. Another issue concerns the measurement of the language ability and social and emotional adjustment constructs. For the language ability constructs, the use of different data sources collected blind for the two language outcomes– ie parent report for the CCC2 and the formal face to face testing of structural language carried out by a member of the team of data collectors in the GUS survey team was considered by the research team to be a benefit, avoiding the risk of reporting bias. But it could be argued that the use of the grammatical elements of the CCC2 might be a better comparison with the pragmatic elements of the same measure. In fact, experience suggests that it is very rare to see the syntactic elements of the CCC2 reported separately and indeed the ability of the different scales of the CCC2 to discriminate at a clinical level has been questioned by the author of the measure. Most researchers focus on the pragmatic elements of the CCC2 which was the main focus of the development of the original measure. In terms of improving the reporting of the results it is important to highlight recent developments in the preregistration of proposed analyses of secondary datasets (106). Although this is more commonly carried out with primary data there is a case for ensuring that this process becomes a feature of future analyses. Finally, the social and emotional adjustment construct assessed using the SDQ covers emotional and behavioural difficulties but not all the positive and negative interactions a child can have. However, the SDQ total score has proven validity and has been shown to correlate strongly with longer and more detailed measures such as the Child Behaviour Checklist at the age measured here (5–6 years) (107). Clearly it is not a diagnostic measure and so findings should be interpreted with caution. However is a valid and reliable indicator of individual differences in the domain of socio-emotional adjustment.

## Conclusions

The results show clearly that parental mental health and HLE in the very early years are associated with structural and pragmatic language outcomes. While the HLE findings do reflect those of other studies, the specific effects of parental mental health, tracking through to pragmatic competence has not previously been demonstrated. The fact that socio- emotional adjustment at school entry is so important in terms of these specific outcomes points to the need for intervention and careful monitoring of children in primary and middle childhood. Services and policy aimed at improving child outcomes through improving home learning environments must go hand in hand with support for the mental health, social- emotional adjustment and wellbeing of parents and children from birth and into the school years.

## Data Availability Statement

Publicly available datasets were analysed in this study. This data can be found here: https://growingupinscotland.org.uk/using-gus-data/data-documentation.

## Ethics Statement

The initial sweep of data collection was subject to medical ethical review by the Scotland A MREC committee (application reference: 04/M RE 1 0/59). Up until and including sweep 8, subsequent annual sweeps have been reviewed *via* substantial amendment submitted to the same committee. Sweep 9 and 10 were subject to ethical review by the NatCen Research Ethics Committee. Written informed consent to participate in this study was provided by the participants' legal guardian/next of kin.

## Author Contributions

JL was the primary author. RR added text, reviewed the manuscript, completed the statistical analysis, and made corrections. CM added text, reviewed the manuscript, and made corrections. NT added text with psychiatric insight, completed the statistical analysis, reviewed the manuscript, and made corrections.

## Funding

The research was funded by the EU's NORFACE Research Programme: Dynamics of Inequality Across the Life-Course NORFACE GRANT 462-16-030, Social InEquality and its Effects on Child Development: A study of birth cohorts in the UK, Germany and the Netherlands.

## Conflict of Interest

The authors declare that the research was conducted in the absence of any commercial or financial relationships that could be construed as a potential conflict of interest.

## Publisher's Note

All claims expressed in this article are solely those of the authors and do not necessarily represent those of their affiliated organizations, or those of the publisher, the editors and the reviewers. Any product that may be evaluated in this article, or claim that may be made by its manufacturer, is not guaranteed or endorsed by the publisher.
