# Supplementary material for: The Role of Social and Emotional Adjustment in Mediating the Relationship Between Early Experiences and Different Language Outcomes
Source: Front Psychiatry. 2021 Dec 2;12:654213. doi: 10.3389/fpsyt.2021.654213 (PMC8674943; doi:10.3389/fpsyt.2021.654213)
Supplement: Supplementary file 1 [file Table_1.docx]

**Supplementary material**

**Supplementary Table 1. Mediated effect of child social and emotional adjustment in the association between parental mental health or home learning environment and child language ability.**

|  |  | Pragmatics |  | Listening comprehension | | | Expressive vocabulary | | |  |
| --- | --- | --- | --- | --- | --- | --- | --- | --- | --- | --- |
|  | β | *95% CI* | *p* | β | *95% CI* | *p* | β | *95% CI* | *p* | |
| Parental Mental Health |  |  |  |  |  |  |  |  |  | |
| Indirect effect | -0.10 | (-0.13, -0.08) | <0.001 | -0.03 | (-0.05, -0.01) | <0.01 | -0.02 | (-0.03, -0.002) | <0.05 | |
| Direct effect | -0.10 | (-0.16, -0.04) | <0.01 | 0.02 | (-0.03, 0.06) | 0.55 | 0.02 | (-0.03, 0.07) | 0.40 | |
| Total effect | -0.20 | (-0.27, -0.14) | <0.001 | -0.01 | (-0.06, 0.03) | 0.57 | 0.008 | (-0.05, 0.05) | 0.87 | |
| Home learning environment | | | | | | | | | | |
| Indirect effect | 0.06 | (0.03, 0.08) | <0.001 | 0.01 | (0.004, 0.02) | <0.01 | 0.01 | (-0.001, 0.02) | 0.08 | |
| Direct effect | 0.06 | (-0.03, 0.14) | 0.18 | 0.15 | (0.07, 0.23) | <0.001 | 0.13 | (0.05, 0.22) | <0.01 | |
| Total effect | 0.11 | (0.03, 0.19) | <0.01 | 0.17 | (0.08, 0.25) | <0.001 | 0.14 | (0.05, 0.22) | <0.01 | |

The estimates correspond in Figure 1 to the direct paths and the indirect paths through child social and emotional adjustment. Each mediation model corresponds to one model. Models are adjusted for maternal education, child sex, birth weight, nonverbal IQ and age at outcome assessment. Models with home learning environment as exposure are further adjusted for parental mental health. Standardized coefficients are presented
